# Associations between endogenous sex hormones and FGF-23 among women and men in the Multi-Ethnic Study of Atherosclerosis

**DOI:** 10.1371/journal.pone.0268759

**Published:** 2022-05-25

**Authors:** Oluseye Ogunmoroti, Olatokunbo Osibogun, Di Zhao, Rupal C. Mehta, Pamela Ouyang, Pamela L. Lutsey, Cassianne Robinson-Cohen, Erin D. Michos

**Affiliations:** 1 Ciccarone Center for the Prevention of Cardiovascular Disease, Johns Hopkins University School of Medicine, Baltimore, Maryland, United States of America; 2 Division of Cardiology, Johns Hopkins University School of Medicine, Baltimore, Maryland, United States of America; 3 Department of Epidemiology, Robert Stempel College of Public Health and Social Work, Florida International University, Miami, Florida, United States of America; 4 Department of Epidemiology, Johns Hopkins University Bloomberg School of Public Health, Baltimore, Maryland, United States of America; 5 Division of Nephrology and Hypertension, Department of Medicine, Northwestern University Feinberg School of Medicine, Chicago, Illinois, United States of America; 6 The Jesse Brown Veterans Administration Medical Center, Chicago, Illinois, United States of America; 7 Division of Epidemiology and Community Health, University of Minnesota School of Public Health, Minneapolis, Minnesota, United States of America; 8 Division of Nephrology, Vanderbilt University Medical Center, Nashville, Tennessee, United States of America; Centro Cardiologico Monzino, ITALY

## Abstract

Elevated levels of testosterone and fibroblast growth factor 23 (FGF-23) are both independently associated with a higher risk of cardiovascular disease (CVD). However, the relationship between sex hormones and FGF-23 is not well established. We explored the association between sex hormones and FGF-23 among middle-aged to older men and women in MESA. We studied 3,052 men and 2,868 postmenopausal women free of CVD at the time of enrollment with baseline serum sex hormones [total testosterone (T), free T, estradiol (E2) and sex hormone binding globulin (SHBG)] and intact FGF-23. In sex-stratified analyses, we examined the cross-sectional associations between log-transformed sex hormones (per 1 SD) and log-transformed FGF-23 using multiple linear regression adjusted for socio-demographics, CVD risk factors, estimated glomerular filtration rate and mineral metabolites (25-hydroxyvitamin D, calcium, phosphorus and parathyroid hormone). The mean (SD) age of study participants was 64 (10) years. The median (IQR) of FGF-23 was similar in women and men [38 (30–46) vs 38 (31–47) pg/mL]. In adjusted analyses, among women, 1 SD increment in free T was associated with 3% higher FGF-23 while SHBG was associated with 2% lower FGF-23. In men, 1 SD increment in E2 was associated with 6% higher FGF-23 whereas total T/E2 ratio was associated with 7% lower FGF-23. In conclusion, this exploratory analysis found that a more androgenic sex hormone profile was directly associated with FGF-23 in women and inversely associated with FGF-23 in men. Longitudinal studies are required to determine whether FGF-23 mediates the relationship between sex hormones and CVD risk.

## Introduction

Fibroblast growth factor-23 (FGF-23) is an endocrine hormone, produced by osteocytes, that exerts its effects on the kidneys by stimulating urinary excretion of phosphate in addition to inhibiting the synthesis as well as promoting the degradation of calcitriol (1, 25-dihydroxyvitamin D3) [1, 2]. In chronic kidney disease (CKD), FGF-23 levels increase as a means to maintain normal serum phosphate homeostasis [3–5]. However, elevated levels of FGF-23 are associated with a higher incidence of cardiovascular disease (CVD) and mortality among CKD patients [1, 6]. Moreover, recent studies have reported an association between elevated FGF-23 levels and a higher risk of incident hypertension, CVD and heart failure (HF) events among people with and without CKD [7–15].

Postmenopausal women with a more androgenic sex hormone profile [higher endogenous free testosterone and lower sex hormone binding globulin (SHBG) levels] have a more adverse cardiovascular phenotype [16]. This phenotype may manifest as greater coronary artery calcium (CAC) progression [17], increased risk for concentric left ventricular remodeling over 10-years [18], greater prevalence of endothelial dysfunction [19], increased aortic stiffness [20] and higher N-terminal pro b-type natriuretic peptide (NT-proBNP) levels [21]. In addition, postmenopausal women with a higher total testosterone/estradiol (T/E2) ratio have an increased risk for incident CVD, coronary heart disease (CHD) and HF events, while higher estradiol is associated with a decreased risk for CHD and HF with reduced ejection fraction [16]. In view of the fact that these associations were either not found or attenuated among men [18, 20], sex differences in endogenous sex hormone profiles may explain the observed differences in cardiovascular outcomes among women and men [22].

Observations from prior animal studies suggest a relationship between sex hormones and FGF-23. For example, FGF-23 levels decreased following orchiectomy of a male rat model from an acute loss of endogenous testosterone (hypo-androgenic state) and testosterone supplementation restored FGF-23 levels [23]. In the female rat model, estrogen treatment resulted in an increase in FGF-23 levels [24]. Likewise, few human studies have shown an association between sex hormones and FGF-23 [25, 26]. However, whether sex hormones interplay with FGF-23 levels to increase CVD risk is still not well established.

Given the sparsity of research showing the link between these two hormones, the aim of this study was to conduct an exploratory analysis of the relationship between sex hormones and FGF-23 among middle-aged to older men and women from a multi-ethnic population free of clinical CVD at baseline and determine whether this relationship is independent of sociodemographic characteristics, CVD risk factors, and other mineral metabolites. We hypothesized that a more androgenic sex hormone profile will be associated with higher levels of FGF-23 among postmenopausal women but not among men of similar age.

## Materials and methods

### Transparency and Openness Policy (TOP)

The Multi-Ethnic Study of Atherosclerosis (MESA) participates in the NIH BioLincc Open program and requests for access to MESA data can be submitted to: https://biolincc.nhlbi.nih.gov/studies/mesa/. Additionally, other researchers can apply to the MESA Coordinating Center to become new investigators after signing a Data Use Agreement (DUA); more details are available at https://www.mesa-nhlbi.org/. Upon receipt of the DUA by the MESA Coordinating Center, access to the de-identified MESA databases would be made available to other researchers with approved proposals. The findings of our study should be easily reproducible through the methods described in this paper.

### Study population

The Multi-Ethnic Study of Atherosclerosis (MESA) is a prospective cohort study of 6,814 women and men without a history of clinical CVD at time of enrollment. Study participants aged 45 to 84 years were recruited from six field centers in the United States (Baltimore, MD; Chicago, IL; Forsyth County, NC; Los Angeles, CA; New York, NY; and St Paul, MN) between the years 2000 and 2002 [27]. MESA examines the characteristics of subclinical CVD and the risk factors that predict progression to clinically overt CVD [27]. The overall cohort had approximately 38% White, 28% Black, 22% Hispanic and 12% Chinese American participants [27].

**Fig 1:**
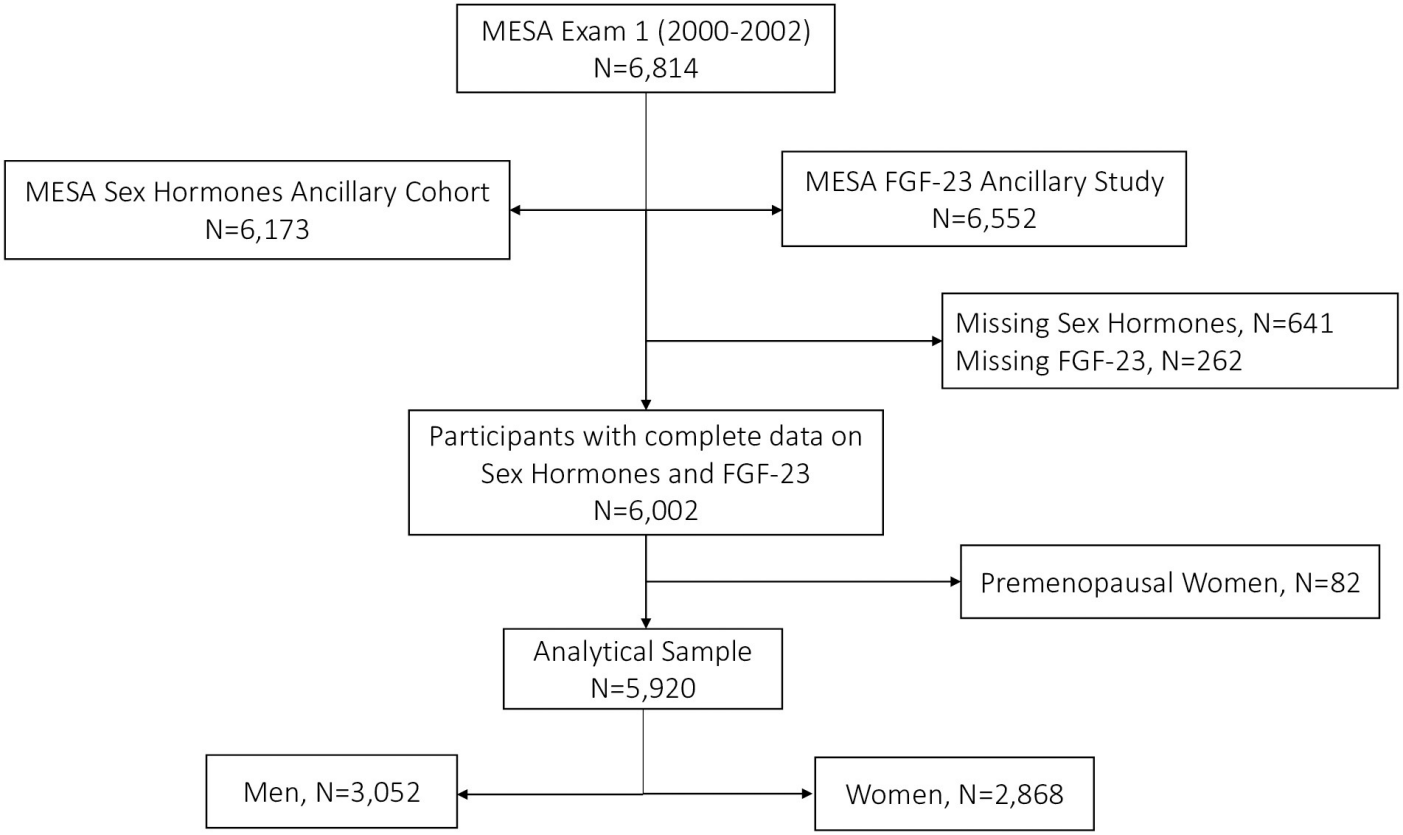
Flowchart of study participants.

Baseline data were used for the present analysis. As shown in the flowchart of study participants in Fig 1, the final analytical sample of this cross-sectional analysis consisted of 5,920 women and men after the exclusion of participants without complete information on sex hormones (n = 641), FGF-23 (n = 262) and premenopausal women (n = 82). We studied only postmenopausal women because sex hormone levels vary between pre- and postmenopausal women. Additionally, the MESA sex hormone ancillary study included only few premenopausal women which limits our ability to properly study this subgroup [19, 21].

### Ethical approval statement

The MESA study protocol was approved by the institutional review boards (IRB) at the aforementioned field centers and written informed consent was given by all participants [27]. At Johns Hopkins University, this study was approved by the Johns Hopkins School of Medicine IRB applicant number NA_00030361.

### Independent variable: Sex hormones

The measurement of sex hormones as reported in previously published research from MESA is described as follows [28–30]. During the baseline examination, MESA study personnel collected fasting blood samples from participants, which were immediately frozen at minus 70°C and shipped to the University of Vermont Core Laboratory for long term storage at minus 80°C. From these stored frozen samples, serum sex hormone levels were later measured at the Steroid Hormone Laboratory of the University of Massachusetts Medical Center (Worcester, MA) as previously described [28]. Total testosterone and dehydroepiandrosterone (DHEA) were measured using radioimmunoassay kits while estradiol was measured using an ultrasensitive radioimmunoassay kit (Diagnostic System Laboratories, Webster, TX) [28]. SHBG was measured with a chemiluminescence enzyme immunometric assay using Immulite kits (Diagnostic Products Corporation, Los Angeles, CA) [28]. Free testosterone was estimated from total testosterone and SHBG using equations derived by Sodergard et al [31]. We calculated the T/E2 ratio by dividing total testosterone by estradiol. The variability of the sex hormone assays was monitored by including approximately 5% blind quality control samples in each batch of samples analyzed [28, 30]. The serum for quality control was obtained from a large pool that was aliquoted into storage vials and assigned labels identical to samples obtained from MESA participants. The intra-assay coefficients of variation for total testosterone, estradiol, DHEA and SHBG were 12.3%, 10.5%, 11.2% and 9.0%, respectively [28].

### Dependent variable: Fibroblast growth factor-23

The measurement of FGF-23 has been previously described in prior research from MESA [8, 13, 32, 33]. As mentioned above, blood samples were collected from study participants during the baseline examination (2000–2002) and stored at -80° at the University of Vermont Laboratory for Clinical Biochemistry using established methods [34], until they were shipped on dry ice in 2011–2012 to the University of Washington where serum FGF-23 levels were measured using the Kainos Immunoassay [35]. This immunoassay detects the full-length biologically intact FGF-23 molecule via mid molecule and distal epitopes. Quality control was monitored using standardized high and low value FGF-23 controls within each run. The coefficient of variation for singular high and low control samples across 81 plates were 6.7% and 12.4%, respectively [13].

### Covariates

MESA study personnel collected data on the covariates included in this study at baseline examination from standardized questionnaires, physical examinations and laboratory testing [27]. We considered sociodemographic factors such as age, race/ethnicity, education and MESA field center. We included CVD risk factors such as smoking (never, former and current), body mass index (BMI) and physical activity (a combination of moderate and vigorous exercises measured in Metabolic Equivalents-minutes per week). Additionally, we included total cholesterol, high-density lipoprotein-cholesterol (HDL-C), systolic blood pressure, diabetes (as defined by the 2003 American Diabetes Association criteria), estimated glomerular filtration rate (eGFR was calculated from the CKD-Epidemiology collaboration equation [36]), use of antihypertensive and lipid-lowering medication. We also considered the related mineral metabolites of 25-hydroxyvitamin D, calcium, phosphorus and parathyroid hormone. For women, we included menopausal variables such as years since menopause and current use of hormone therapy.

### Statistical analysis

We stratified all analyses by sex because of the differences in sex hormones between women and men. We reported continuous variables as means with standard deviation (SD) or medians with interquartile range (IQR) for skewed distributions. Additionally, we reported categorical variables as frequencies with percentages. We logarithmically transformed the sex hormones and FGF-23 because of their skewed distributions and modeled both as continuous variables. The sex hormones and T/E2 ratio expressed per 1 SD were the independent variables and FGF-23 was the dependent variable. Using multiple linear regression models, we reported the exponentiated beta-coefficients with 95% confidence intervals of the association between each sex hormone and FGF-23 as well as the association between the T/E2 ratio and FGF-23. We presented four progressively adjusted models. Model 1 was adjusted for age, race/ethnicity and MESA field center. Model 2 was adjusted for education and CVD risk factors such as smoking, BMI and physical activity. In model 3, we adjusted for additional CVD risk factors such as total cholesterol, HDL-C, systolic blood pressure, diabetes, eGFR, use of antihypertensive and lipid-lowering medications. For women, we also adjusted for years since menopause and current use of hormone therapy in model 3. The final model (model 4) was additionally adjusted for the related mineral metabolites of 25-hydroxyvitamin D, calcium, phosphorus and parathyroid hormone.

Furthermore, we used restricted cubic splines adjusted for model 4 covariates to assess for non-linear relationships between logarithmically transformed sex hormones and the differences in logarithmically transformed FGF-23. In separate analysis for women and men, we tested whether race/ethnicity was an effect modifier of the association between sex hormones and FGF-23 by including interaction terms in the fully adjusted model. Where interaction was significant, we performed stratified analysis by subgroups. Lastly, we conducted a sensitivity analysis among women stratified by current use of hormone therapy and we adjusted for model 4 covariates. We performed all our analyses using STATA version 15.0 (StataCorp LP, College Station, Texas). In view of the higher probability of false positive findings from multiple comparisons, a Bonferroni corrected two-sided P-value <0.0021 [0.05 divided by 24 (6 exposure variables x 1 outcome variable x 4 models)] was considered statistically significant.

## Results

The baseline characteristics of study participants (N = 5,920) stratified by sex are presented in Table 1. Women comprised 48% of participants with a mean age (SD) of 65 (9) years compared to 62 (10) years for men. The median (IQR) of FGF-23 was 38 (30–46) in women and 38 (31–47) pg/mL in men. Approximately 32% of women reported current use of hormone therapy. A smaller proportion of women had a bachelor’s degree. Women had higher BMI, systolic blood pressure and total cholesterol. A larger proportion of men were smokers, diabetic and physically active. Total testosterone, free testosterone, DHEA and T/E2 ratio were higher in men but SHBG was higher in women. Estradiol and FGF-23 levels were similar in women and men.

**Table 1:** Baseline characteristics of study participants, MESA 2000–2002.

	Total N = 5,920	Women n = 2,868	Men n = 3,052
Age, years	64 (10)	65 (9)	62 (10)
Race/ethnicity			
White	2,304 (39%)	1,096 (38%)	1,208 (40%)
Chinese American	727 (12%)	341 (12%)	386 (13%)
Black	1,583 (27%)	805 (28%)	778 (25%)
Hispanic	1,306 (22%)	626 (22%)	680 (22%)
Education			
≥ bachelor’s degree	2,063 (35%)	804 (28%)	1,259 (41%)
< bachelor’s degree	3,835 (65%)	2,053 (72%)	1,782 (59%)
Smoking status			
Current smoker	742 (13%)	311 (11%)	431 (14%)
Former smoker	2,226 (38%)	862 (30%)	1,364 (45%)
Never smoker	2,931 (50%)	1,684 (59%)	1,247 (41%)
Diabetes mellitus	771 (13%)	352 (12%)	419 (14%)
Use of antihypertensive medication	2,276 (38%)	1,201 (42%)	1,075 (35%)
Use of lipid-lowering medication	1,037 (18%)	537 (19%)	500 (16%)
BMI, kg/m^2^	28 (5)	29 (6)	28 (4)
Physical activity, MET-minutes/week	3,960 (1,950–7,425)	3,540 (1,740–6,495)	4,470 (2,190–8,475)
Systolic blood pressure, mmHg	128 (22)	130 (23)	126 (19)
eGFR, ml/min per 1.73m^2^	77 (16)	75 (16)	78 (16)
Total cholesterol, mg/dL	195 (36)	201 (36)	188 (35)
HDL-C, mg/dL	51 (15)	57 (15)	45 (12)
*Current use of HT	914 (33%)	914 (33%)	-
*Years since menopause, years	16 (8–25)	16 (8–25)	-
Total testosterone, nmoI/L	6.8 (0.9–14.4)	0.9 (0.6–1.3)	14.2 (11.3–17.8)
Free testosterone, percent	1.7 (1.2–2.1)	1.3 (0.9–1.7)	2.0 (1.7–2.3)
Estradiol, nmoI/L	0.1 (0.06–0.14)	0.1 (0.05–0.16)	0.1 (0.09–0.14)
DHEA, nmoI/L	11 (8–16)	10 (7–15)	13 (9–17)
Sex Hormone Binding Globulin, nmoI/L	47 (34–68)	60 (41–95)	41 (31–53)
Total Testosterone: Estradiol ratio	61 (12–132)	12 (5–22)	128 (93–174)
FGF-23, pg/mL	38 (31–47)	38 (30–46)	38 (31–47)
25-hydroxyvitamin D, ng/mL	21 (15–30)	21 (14–29)	22 (15–30)
Calcium, mg/dL	9.6 (9.4–9.9)	9.7 (9.4–9.9)	9.6 (9.4–9.8)
Phosphorus, mg/dL	3.7 (3.3–4.0)	3.9 (3.5–4.2)	3.5 (3.2–3.8)
Parathyroid hormone, pg/mL	41 (31–54)	42 (32–55)	40 (31–52)

Abbreviations: BMI, Body mass index; DHEA, Dehydroepiandrosterone; eGFR, Estimated glomerular filtration rate; FGF-23, Fibroblast growth factor-23; HDL-C, High-density lipoprotein cholesterol; HT, Hormone therapy; MESA, Multi-Ethnic Study of Atherosclerosis; MET, Metabolic equivalent of task. Data were presented as mean (SD) or n (%) or median (IQR).

*Data was collected only in women.

The adjusted cross-sectional associations between sex hormones and FGF-23 in women are shown in Table 2. In women, 1 SD increment in free testosterone was independently associated with 5% higher levels of FGF-23 [Percent difference: 5% (95% CI: 3%, 6%)] but 1 SD increment in SHBG was independently associated with 4% lower levels of FGF-23 [-4% (-5%, -3%)], after adjusting for demographic variables (model 1). These associations remained after further adjusting for CVD risk factors, years since menopause, current use of hormone therapy and related mineral metabolites of 25-hydroxyvitamin D, calcium, phosphorus and parathyroid hormone in model 4 [free testosterone: 3% (1%, 5%), SHBG: -3% (-4%, -1%)]. In the sensitivity analysis stratified by women who were currently on hormone therapy and women who were not on hormone therapy, we found no statistically significant associations between sex hormones and FGF-23 (S1 Table).

**Table 2:** Percent difference with 95% CI of the associations between log-transformed endogenous sex hormones and FGF-23 among women, n = 2,868.

Sex hormones (log-transformed) per 1SD	Percent difference (95% CI) for log (FGF-23)			
	Model 1	Model 2	Model 3	Model 4
Total testosterone (nmoI/L)	1.0 (-2.0, 4.0)	0.0 (-3.0, 3.0)	-1.0 (-4.0, 2.0)	-1.0 (-4.0, 2.0)
Free Testosterone (Percent)	**5.0 (3.0, 6.0)** *	**4.0 (2.0, 5.0)** *	**3.0 (1.0, 5.0)** *	**3.0 (1.0, 5.0)** *
Estradiol (nmoI/L)	0.0 (-2.0, 1.0)	-1.0 (-2.0, 0.0)	0.0 (-1.0, 1.0)	1.0 (-1.0, 2.0)
DHEA (nmoI/L)	1.0 (-1.0, 2.0)	1.0 (-1.0, 2.0)	0.0 (-1.0, 2.0)	0.0 (-1.0, 2.0)
Sex Hormone Binding Globulin (nmoI/L)	**-4.0 (-5.0, -3.0)** *	**-3.0 (-5.0, -2.0)** *	**-3.0 (-4.0, -1.0)** *	**-3.0 (-4.0, -1.0)** *
Total Testosterone: Estradiol ratio	1.0 (-1.0, 3.0)	1.0 (0.0, 3.0)	-1.0 (-3.0, 2.0)	-1.0 (-3.0, 1.0)

Abbreviation: DHEA, Dehydroepiandrosterone; FGF, Fibroblast growth factor.

Intact FGF-23 was the dependent variable and was log-transformed for the analysis. The sex hormones were the independent variables and were log-transformed and modeled separately per 1 standard deviation.

We presented results as percent difference with 95% confidence interval calculated from [Exp (β) -1]*100 reflecting the percent difference of the geometric mean of FGF-23.

*Statistically significant results at Bonferroni corrected P <0.0021 are in bold font.

^†^Statistical significant results at P <0.05 are in italics.

Model 1: adjusted for age, race/ethnicity and MESA field center.

Model 2: model 1 plus smoking, body mass index, education and physical activity

Model 3: model 2 plus total cholesterol, high-density lipoprotein cholesterol, use of lipid-lowering medication, systolic blood pressure, use of antihypertensive medication, diabetes and estimated glomerular filtration rate.

In women, we additionally adjusted model 3 for current use of hormone therapy and years since menopause.

Model 4: model 3 plus the related mineral metabolites of 25-hydroxyvitamin D, calcium, phosphorus and parathyroid hormone.

**Fig 2:**
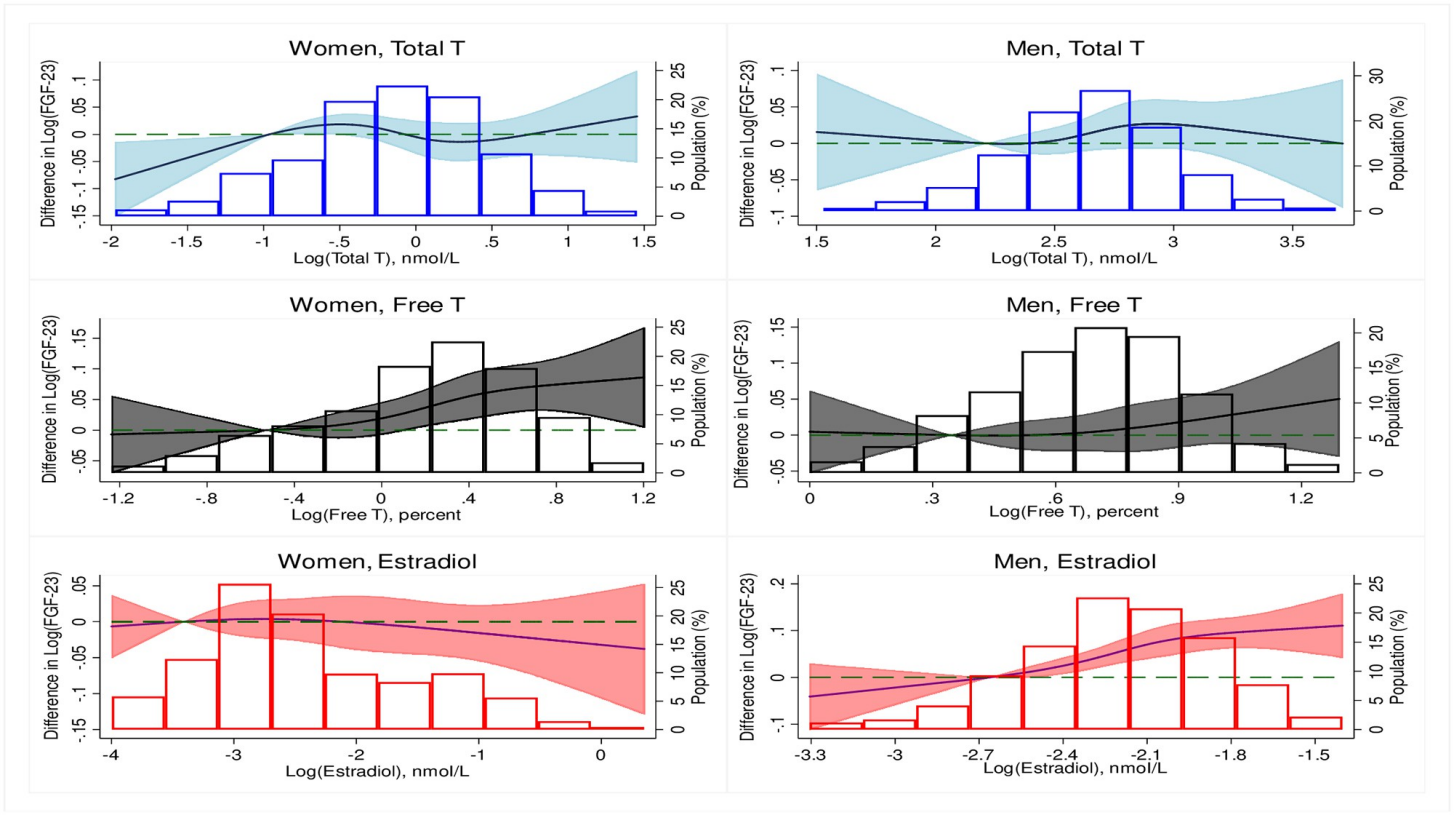
Restricted cubic splines of the associations between logarithmically transformed sex hormones (Total T, Free T and Estradiol) and FGF-23. [The model was adjusted for demographic variables, CVD risk factors and related mineral metabolites of 25-hydroxyvitamin D, calcium, phosphorus and parathyroid hormone (In women, the model was additionally adjusted for years since menopause and current use of hormone therapy)]. Difference in logarithmically transformed FGF-23 and proportion of the population are on the y-axis while logarithmically transformed sex hormones are on the x-axis. **Abbreviation**: T, testosterone.

**Fig 3:**
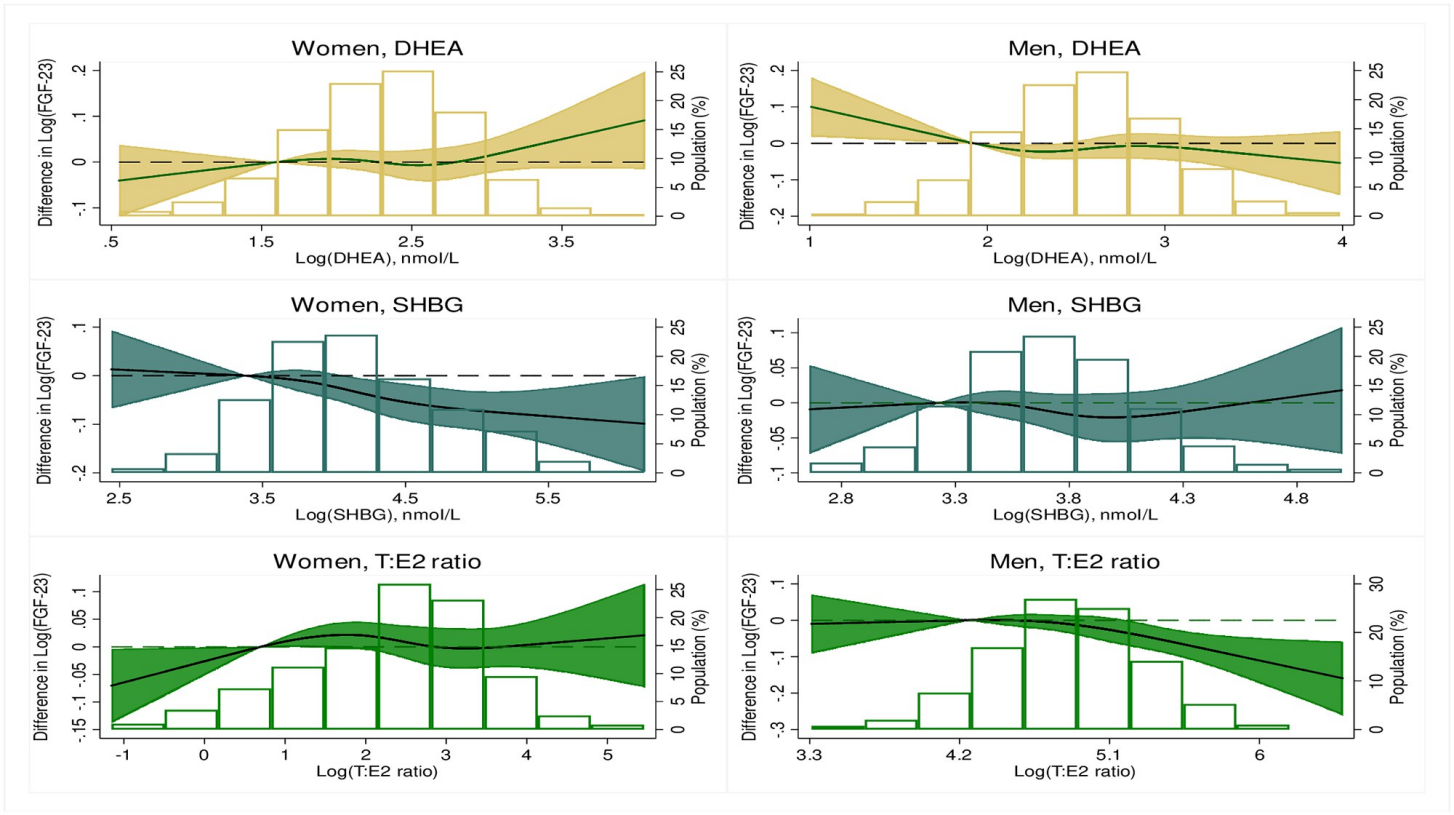
Restricted cubic splines of the associations between logarithmically transformed sex hormones (DHEA, SHBG and T/E2 ratio) and FGF-23. [The model was adjusted for demographic variables, CVD risk factors and related mineral metabolites of 25-hydroxyvitamin D, calcium, phosphorus and parathyroid hormone (In women, the model was additionally adjusted for years since menopause and current use of hormone therapy)]. Difference in logarithmically transformed FGF-23 and proportion of the population are on the y-axis while logarithmically transformed sex hormones are on the x-axis. **Abbreviations**: DHEA, dehydroepiandrosterone; SHBG, sex hormone binding globulin; T: E2 ratio, Testosterone: Estradiol ratio.

Table 3 shows the adjusted cross-sectional associations between sex hormones and FGF-23 in men. One SD increment in SHBG and T/E2 ratio were independently associated with 4% and 11% lower levels of FGF-23 in model 1 [-4% (-6%, -2%) and -11% (-14%, -7%), respectively]. Additionally, 1 SD increment in free testosterone and estradiol were associated with 5% and 7% higher levels of FGF-23 [5% (3%, 8%) and 7% (5%, 10%), respectively]. However, in the fully adjusted model (model 4), the associations remained significant for only estradiol and T/E2 ratio [6% (4%, 9%) and -7% (-10%, -3%), respectively]. In the restricted cubic splines (Figs 2 and 3), we found that the relationship between the logarithmically transformed sex hormones and FGF-23 was mostly non-linear in both women and men. We found significant interaction by race/ethnicity at P <0.05 for only the association between DHEA and FGF-23 in women (P = 0.042). However, the results of the stratified analysis by race/ethnicity did not show any significant associations: White participants, -1 (-3, 1); Chinese-American participants, 2 (-2, 6); Black participants, 1 (-2, 4); Hispanic participants, 1 (-1, 4).

**Table 3:** Percent difference with 95% CI of the associations between log-transformed endogenous sex hormones and FGF-23 among men, n = 3,052.

Sex hormones (log-transformed) per 1SD	Percent difference (95% CI) for log (FGF-23)			
	Model 1	Model 2	Model 3	Model 4
Total testosterone (nmoI/L)	*-4*.*0 (-8*.*0*, *0*.*0)*†	-1.0 (-5.0, 3.0)	-1.0 (-5.0, 3.0)	1.0 (-3.0, 6.0)
Free Testosterone (Percent)	**5.0 (3.0, 8.0)** *	**4.0 (1.0, 6.0)** *	1.0 (-1.0, 3.0)	1.0 (-1.0, 3.0)
Estradiol (nmoI/L)	**7.0 (5.0, 10.0)** *	**7.0 (4.0, 9.0)** *	**6.0 (4.0, 8.0)** *	**6.0 (4.0, 9.0)** *
DHEA (nmoI/L)	*-2*.*0 (-3*.*0*, *0*.*0)*†	-1.0 (-3.0, 0.0)	-1.0 (-3.0, 0.0)	-1.0 (-3.0, 0.0)
Sex Hormone Binding Globulin (nmoI/L)	**-4.0 (-6.0, -2.0)** *	*-3*.*0 (-5*.*0*, *-1*.*0)*†	-1.0 (-3.0, 1.0)	-1.0 (-3.0, 1.0)
Total Testosterone: Estradiol ratio	**-11.0 (-14.0, -7.0)** *	**-9.0 (-12.0, -6.0)** *	**-8.0 (-11.0, -4.0)** *	**-7.0 (-10.0, -3.0)** *

Abbreviation: DHEA, Dehydroepiandrosterone; FGF, Fibroblast growth factor.

Intact FGF-23 was the dependent variable and was log-transformed for analysis. The sex hormones were the independent variables and were log-transformed and modeled separately per 1 standard deviation.

We presented results as percent difference with 95% confidence interval calculated from [Exp (β) -1]*100 reflecting the percent difference of the geometric mean of FGF-23.

*Statistically significant results at Bonferroni corrected P <0.0021 are in bold font.

^†^Statistically significant results at P <0.05 are in italics.

Model 1: adjusted for age, race/ethnicity and MESA field center.

Model 2: model 1 plus smoking, body mass index, education and physical activity

Model 3: model 2 plus total cholesterol, high-density lipoprotein cholesterol, use of lipid-lowering medication, systolic blood pressure, use of antihypertensive medication, diabetes and estimated glomerular filtration rate.

Model 4: model 3 plus the related mineral metabolites of 25-hydroxyvitamin D, calcium, phosphorus and parathyroid hormone.

## Discussion

In this exploratory analysis of a large multi-ethnic cohort of postmenopausal women and men free of clinical CVD at baseline, we found that among women, higher levels of free testosterone were independently associated with higher FGF-23 and higher levels of SHBG were associated with lower FGF-23. Since free testosterone reflects testosterone that is unbound to SHBG and albumin, the relationships of free testosterone and SHBG with FGF-23 in opposite directions are consistent. In men, higher levels of estradiol were associated with higher FGF-23 and higher levels of T/E2 ratio were associated with lower FGF-23. These aforementioned relationships were all independent of CVD risk factors and other markers of mineral metabolism such as serum 25-hydroxyvitamin D, calcium and phosphate, which are influenced by FGF-23 levels.

The few studies that have examined the relationship between sex hormones and FGF-23 reported conflicting findings [23–26, 37]. Burnett-Bowie and colleagues in their study on the effects of gonadal steroid withdrawal on serum phosphate and FGF-23 levels in men found that after the administration of goserelin acetate, a gonadotropin releasing hormone (GnRH) analog, mean serum testosterone and estradiol decreased resulting in an increase in serum phosphate levels but no change in FGF-23 levels [25]. In contrast to the results of this study, our findings showed that in men, higher estradiol levels were associated with higher levels of FGF-23. Of note, the molecular mechanisms driving these associations are still under investigation [25]. In another study of the effect of testosterone replacement therapy on vitamin D and FGF-23 levels in congenital hypogonadism, Haymana and colleagues observed that transdermal testosterone replacement therapy increased plasma FGF-23 after approximately 4 months of follow-up in young treatment naive men [26]. However, our study findings do not provide sufficient evidence of a direct or inverse relationship between total testosterone and FGF-23.

Saki and colleagues in an experimental study investigating the effect of testosterone and letrozole on 1, 25-dihydroxyvitamin D and FGF-23 in male rats reported a significant reduction in 1, 25-dihydroxyvitamin D and FGF-23 in orchiectomized rats compared to the control group [23]. In addition, they observed a correction of 1, 25-dihydroxyvitamin D and FGF-23 levels after the orchiectomized rats were treated with testosterone [23]. Consistent with this animal model, we found that a more androgenic sex hormone profile (higher free testosterone, lower SHBG) was associated with higher FGF-23 levels in women, but not in men. As postulated by Saki and colleagues, it is likely that testosterone directly increases serum FGF-23 independent of its aromatization to estradiol or indirectly through the upregulation of the FGF-23 gene by 1, 25-dihydroxyvitamin D [23, 38, 39].

Furthermore, an experimental study conducted by Carrillo-Lopez and colleagues to investigate the factors and mechanisms involved in the effect of estrogens on the parathyroid gland found that female rats treated with estrogen had significantly higher serum and bone FGF-23 levels as well as significantly lower serum calcitriol, phosphorus and parathyroid hormone levels [24]. In our study, we found no association between serum estradiol levels and FGF-23 levels in women, although estradiol levels were generally low in these postmenopausal women. However, higher estradiol levels were associated with higher FGF-23 levels in men. In contrast to the findings from Carrillo-Lopez and colleagues, a cross-sectional study conducted by Ix and colleagues [37] to examine the link between exogenous estrogen therapy and FGF-23, reported that FGF-23 levels were significantly higher in older women not on estrogen therapy compared to women on estrogen therapy or men. In our study, FGF-23 levels were only slightly higher in women not currently on hormone therapy [38 (31, 47) pg/mL] compared to the levels for women currently on hormone therapy [36 (29, 45) pg/mL].

Given that the relationship between endogenous sex hormones and FGF-23 from population studies has not been previously well established, we undertook this study to better understand the potential mechanism that might explain why both sex hormone profiles (pro-androgenic pattern in women, hypo-androgenic pattern in men) and FGF-23 are associated with elevated CVD risk [14, 16]. However, the clinical implication of the relationships we describe is unclear. Even if sex hormones did influence CVD risk via elevated FGF-23 levels, it is unclear what the best interventions should be to modify sex hormone levels for risk reduction. Randomized clinical trials have failed to demonstrate a CVD benefit of hormone therapy in post-menopausal women [40–42].

This study has some strengths. We used data from a large, diverse, community-based population of women and men who did not have clinical CVD at the time of study enrollment. The availability of data on a large number of variables with standardized measurements enabled us to examine five different sex hormones and reduce the effect of potential confounding factors. We were also able to account for other important mineral metabolite markers. Nevertheless, our study has some limitations. We cannot prove causality or show whether a temporal relationship exists between sex hormones and FGF-23 because this study was observational with a cross-sectional design. In addition, residual confounding may still be present even though we accounted for numerous confounding factors in our analysis. We used only one baseline measurement of sex hormones to determine the hormonal status of study participants instead of serial measurements, although previous studies suggest a single assessment of sex hormones is reliable [43, 44]. Lastly, sex hormones in MESA were measured by radioimmunoassay and not by mass spectrometry which is the current gold standard. The accuracy of the equations used to estimate free testosterone have been questioned in the literature [45]; therefore we reported results for both measured total testosterone as well as estimated free testosterone. We chose to present the results for free testosterone because it is more strongly linked with cardiovascular risk in women than total testosterone [16, 17, 19–21].

## Conclusions

In this exploratory analysis, we found that a more androgenic sex hormone profile was directly associated with FGF-23 in women while inversely associated with FGF-23 in men. In light of the fact that CVD is the leading cause of morbidity and mortality globally, a thorough understanding of the different biological pathways underlying the incidence of CVD is of considerable importance to facilitate the development of improved prevention and therapeutic options [46]. There is a need for well-designed prospective studies to clarify the conflicting findings from prior studies and determine if a causal relationship exists between sex hormones and FGF-23. In addition, these new studies should investigate whether FGF-23 acts as a mediator in the relationship between sex hormones and CVD risk.

## Supporting information

S1 Table Percent difference with 95% CI of the associations between log-transformed endogenous sex hormone and FGF-23 among women stratified by use of hormone therapy. (DOCX)
