# Supplementary material for: Associations between endogenous sex hormones and FGF-23 among women and men in the Multi-Ethnic Study of Atherosclerosis
Source: PLoS One. 2022 May 25;17(5):e0268759. doi: 10.1371/journal.pone.0268759 (PMC9132299; doi:10.1371/journal.pone.0268759)
Supplement: S1 Table — (DOCX) [file pone.0268759.s001.docx]

**Supporting Information**

| Sex hormones  (log-transformed) per 1SD | Percent difference (95% CI) for log (FGF-23) | |
| --- | --- | --- |
|  | Currently on hormone therapy  n=914 | Not currently on hormone therapy  n=1,887 |
| Total testosterone (nmoI/L) | 2.0 (-3.0, 7.0) | -2.0 (-6.0, 1.0) |
| Free Testosterone (Percent) | *4.0 (1.0, 6.0)** | 2.0 (0.0, 5.0) |
| Estradiol (nmoI/L) | 0.0 (-2.0, 2.0) | 1.0 (-1.0, 3.0) |
| DHEA (nmoI/L) | 1.0 (-2.0, 3.0) | 1.0 (-1.0, 2.0) |
| Sex Hormone Binding Globulin (nmoI/L) | *-4.0 (-6.0, -1.0)** | -2.0 (-4.0, 0.0) |
| Total Testosterone: Estradiol ratio | 1.0 (-2.0, 5.0) | -3.0 (-6.0, 0.0) |
| Abbreviation: DHEA, Dehydroepiandrosterone; FGF, Fibroblast growth factor.  Intact FGF-23 was the dependent variable and was log-transformed for the analysis. The sex hormones were the independent variables and were log-transformed and modeled separately per 1 standard deviation.  We presented results as percent difference with 95% confidence interval calculated from [Exp (β) -1]*100 reflecting the percent difference of the geometric mean of FGF-23.  *Statistically significant results at P<0.01 are in italics but not significant at Bonferroni correction of P<0.0021.  The model was adjusted for age, race/ethnicity, MESA field center, smoking, body mass index, education, physical activity, total cholesterol, high-density lipoprotein cholesterol, use of lipid-lowering medication, systolic blood pressure, use of antihypertensive medication, diabetes, estimated glomerular filtration rate, related mineral metabolites of 25-hydroxyvitamin D, calcium, phosphorus and parathyroid hormone. | | |

**S1 Table. Percent difference with 95% CI of the associations between log-transformed endogenous sex hormones and FGF-23 among women stratified by use of hormone therapy.**
